# Recombinant VSVs: A Promising Tool for Virotherapy

**DOI:** 10.32607/actanaturae.27501

**Published:** 2024

**Authors:** K. A. Vorona, V. D. Moroz, N. B. Gasanov, A. V. Karabelsky

**Affiliations:** Sirius University of Science and Technology, Krasnodar Region, Sirius Federal Territory, 354340 Russian Federation

**Keywords:** oncolytic viruses, vesicular stomatitis virus, cancer immunotherapy, interferon-stimulated gene, biomarker of resistance

## Abstract

Cancer is one of the leading causes of death worldwide. Traditional cancer treatments include surgery, radiotherapy, and chemotherapy, as well as combinations of these treatments. Despite significant advances in these fields, the search for innovative ways to treat malignant tumors, including the application of oncolytic viruses, remains relevant. One such virus is the vesicular stomatitis virus (VSV), which possess a number of useful oncolytic properties. However, VSV-based drugs are still in their infancy and are yet to be approved for clinical use. This review discusses the mechanisms of oncogenesis, the antiviral response of tumor and normal cells, and markers of tumor cell resistance to VSV virotherapy. In addition, it examines methods for producing and arming recombinant VSV and provides examples of clinical trials. The data presented will allow better assessment of the prospects of using VSV as an oncolytic.

## INTRODUCTION

Cancer is one of the leading causes of death in developed countries. Over the past years, the application of immunotherapy strategies in clinical practice has improved treatment effectiveness for many cancers. Unfortunately, positive outcomes have been achieved in only a limited type of malignant tumors which account for approximately one-third of all cases. Furthermore, not all patients appear to respond to therapy [1, 2].

It is anticipated that obstacles to the treatment of malignant tumors can be bypassed using oncolytic viruses (oncolytics). These viruses are capable of specifically replicating in cancer cells while remaining safe for the organism [3]. The viruses can replicate in cancer cells due to the impaired antiviral response associated with dysfunctional interferon (IFN) production. Interferons inhibit viral replication as well as the formation and spread of virus particles by activating signaling pathways that slow down metabolism in infected and neighboring cells. Oncolytic viruses are highly specific to cancer cells with a restricted IFN response; they induce an inflammatory response in the tumor and fine-tune the immune system to target the inflammation site, whereas in healthy cells, viruses are destroyed due to IFN-mediated immune responses.

The vesicular stomatitis virus (VSV), a negative- sense RNA virus belonging to the family* Rhabdoviridae*, is one of the promising oncolytic viruses [4]. VSV has a number of advantages: it is not integrated into the host genome and has a broad tropism; its genome can be relatively easily modified, and a very small percentage of people are seropositive for VSV [5].

Recombinant VSV (rVSV)-based drugs are being investigated *in vitro *and *in vivo *[6, 7, 8, 9, 10]; clinical trials to assess their effectiveness in the treatment of colorectal cancer, melanoma, lung cancer, breast cancer, malignant lymphoma, and other cancers are ongoing (NCT02923466, NCT04046445, NCT04291105, NCT03017820, NCT03865212, NCT04291105, NCT03120624, NCT03456908, NCT05846516, NCT05644509, and NCT01042379).

Research into the oncolysis mechanisms, markers, and signaling pathways responsible for the resistance of cancer and healthy cells to the virus can explain the variability in the response of different tumors to virotherapy and allow one to find ways to optimize recombinant therapeutic VSVs [11].

## THE PROBLEM OF CANCER AND ONCOGENESIS

Approximately one in five people develops cancer throughout their lifetime; cancer-related deaths have been documented in almost one in nine men and one in twelve women. The most common types of cancer (> 60% of all cancer cases) include lung cancer, breast cancer, colorectal cancer, prostate cancer, stomach cancer, liver cancer, thyroid cancer, cervical cancer, bladder cancer, and non-Hodgkin lymphoma [12, 13].

D. Hanahan [14] described the key characteristics of malignant tumors. They include eight hallmarks: the ability to evade the impact of oncosuppressors; resistance to apoptosis; the ability to sustain proliferative signaling and induce angiogenesis; invasion and metastasis; replicative immortality; and immune evasion and alteration of cellular metabolism. The emergence of tumor cells is associated with genomic instability and the accumulation of mutations altering the cell morphology and function, as well as with epigenetic reprogramming of cell identity and chronic inflammation.

At the molecular level, carcinogenesis is caused by mutations in oncogenes and tumor suppressor genes. Mutations in proto-oncogenes may promote their transformation into oncogenes, in turn inducing the synthesis of oncoproteins, which enhance cell proliferation and promote the evasion of apoptosis [15, 16]. On the other hand, suppressor genes encode functional proteins that inhibit the oncogenic transformation of cells, including factors controlling cell division, cell death, and DNA repair. Mutations in tumor suppressor genes lead to inactivation of their products and, therefore, tumor development [15, 16, 17]. In addition, there is a growing body of evidence indicating other potential reasons for cancer development. Thus, epigenetic changes may contribute to the development of the main characteristics of tumor. Changes in the epigenetic DNA profile in tumor cells are associated with hypoxia caused by insufficient vascularization of tissues and cells, which leads to reduced activity of TET demethylases, resulting in significant changes in the methylome, and DNA hypermethylation in particular [14, 18]. Chronic inflammation can be another reason behind tumor growth induction [19]. Chronic inflammation processes can be induced, and the risk of cancer development or progression can be increased by *Helicobacter pylori* in patients with stomach cancer and MALT lymphoma, by the papillomavirus and hepatitis virus in patients with cervical and liver cancer, respectively, by autoimmune diseases (e.g., inflammatory bowel disease in patients with colorectal cancer), and by an inflammation of unknown origin (e.g., prostatitis in patients with prostate cancer) [20].

## CANCER VIROTHERAPY

Surgical intervention, radiation therapy and chemotherapy, as well as their combinations, are still the key strategies used in cancer treatment. However, poor treatment effectiveness at late stages and the high risk of recurrence necessitate a search for innovative methods. Thus, cytokines activating immune cells [21, 22], adoptive cell therapy (CAR-T therapy) [23, 24, 25], immunotherapy based on antibodies (immune checkpoint inhibitors or antibody drug conjugates) [26, 27], antitumor vaccines, etc. [28, 29, 30] are used in cancer immunotherapy. In recent years, immunotherapy methods increasing the treatment effectiveness in some cancers are moving ever closer to clinical practice, but not all patients respond to therapy [1].

The main reasons for the lack of response to immunotherapy include the insufficient immunogenicity of cancer cells as well as challenges related to the delivery of immunocompetent cells and immunotherapeutic agents to their targets [2]. These hurdles can be overcome by using oncolytic viruses, a new class of antitumor agents promoting tumor regression through the preferential replication of viruses in cancer cells, induction of immunogenic apoptosis, and stimulation of antitumor immunity [3]. Oncolytic viruses display enhanced tropism for tumors where the dysfunction of antiviral response factors allows viruses to preferentially replicate in cancer cells [31].

Several drugs based on oncolytic viruses have been approved for cancer treatment worldwide. In 2004, the State Agency of Medicines of the Republic of Latvia approved Rigvir for the virotherapy of melanoma. Rigvir is derived from the native strain of echovirus serotype 7 (ECHO-7), a nonpathogenic intestinal cytopathic RNA enterovirus belonging to the family *Picornaviridae*. However, Rigvir production was suspended in 2019 because of violations of the manufacturing process and quality control standards [3, 32]. In 2006, the use of oncolytic virus H101, a genetically modified adenovirus, in combination with cytotoxic chemotherapy, was approved for the treatment of head and neck cancer in China [3, 33]. In 2015, the U.S. Food and Drug Administration (FDA) approved Talimogene Laherparepvec (T-VEC), an attenuated herpes simplex virus type 1 (HPV-1) encoding the granulocyte-macrophage colony-stimulating factor (GM-CSF), for topical treatment of inoperable dermal, subcutaneous, and nodular lesions in patients with recurrent melanoma after primary surgery [3, 32, 34]. The effectiveness and safety of T-VEC were studied in a multicenter randomized clinical trial; afterwards, the drug was approved in Europe, Australia, and Israel. Recent clinical trials have demonstrated that a combination of oncolytic viruses and immune checkpoint blockade improves the therapeutic response [35, 36].

**Antiviral response in normal and cancer cells**

**Fig. 1:**
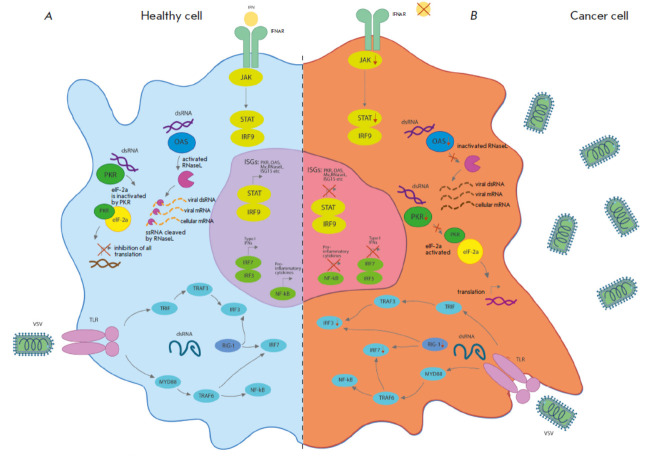
Oncolytic viruses in tumor cells with defective antiviral responses. (*A*) During viral infection, most normal cells activate an antiviral mechanism that can be triggered by PAMPs associated with the viral pathogen or by detection of viral nucleic acids. TLRs transmit signals through MYD88, inducing the production of pro-inflammatory cytokines and interferons that activate the JAK-STAT signaling pathway; (*B*) the cancer cell response to a viral infection is altered. In cancer cells, the activity of critical components of the innate signaling pathway, including RIG-I, IRF7, and IRF3, can be suppressed, thus limiting the detection of virus particles and making cancer cells more susceptible to viral replication. Additionally, critical components of the IFN signaling pathway can be inhibited in cancer cells [39]

Innate immunity is the body’s defense system against foreign and potentially harmful pathogens that exists before the initial entry of pathogens into the body [37, 38]. In healthy cells, various signaling pathways are activated in response to a viral infection (*Fig. 1*), which can be stimulated by a local release of type I interferon (IFN-I) or the activation of intracellular Toll-like receptors (TLRs). TLRs recognize evolutionarily conserved pathogen-associated molecular patterns (PAMPs), which may include elements of viral origin (capsids, DNA, RNA, and proteins). TLR signaling activates host cell’s antiviral responses and systemic innate immunity. Several host cell factors such as TRAF3 (TNF receptor-associated factor 3), IRF3 (IFN regulatory factor 3), IRF7 (IFN regulatory factor 7), and RIG-I (retinoic acid-inducible gene I) were found to play an important role in halting viral replication and reducing viral infectivity. These factors activate the JAK-STAT pathway coordinating the antiviral response in infected cells [39].

In response to virus entry, interferon production is also activated in cells. There are three types of interferons: type I interferons (IFN-I): IFN-α, IFN-β, and IFN-ω; type II interferons (IFN-II): IFN-γ; and type III interferons (IFN-III): IFN-λ1, IFN-λ2, IFN-λ3 (also known as IL29, IL28A, and IL28B, respectively), and IFN-λ4 [40, 41]. Interferons inhibit viral replication, the formation of virus particles, and virion spread both in the infected cell and in neighboring cells by activating signaling cascades that slow down metabolism. Interferons enhance the synthesis of the major histocompatibility complex classes I and II (MHC-I, MHC-II) molecules and stimulate the activity of immunoproteasomes. The elevated MHC-I level promotes efficient presentation of viral peptides by cytotoxic T lymphocytes and killer cells. The immunoproteasome performs the proteolysis of viral peptides, which are then transported to the endoplasmic reticulum and are presented as part of MHC class I. The high MHC-II level ensures that viral antigens are presented by T-helper cells, which in response secrete cytokines regulating the rest of the immune system. Meanwhile, interferons reduce cell proliferation and activate p53 proapoptotic protein [42].

IFN-I activates the IFNAR (type I IFN receptor) complex, which involves the IFNAR1 and IFNAR2 subunits. IFN-I is essential for eliciting a robust antiviral response. Mice lacking IFNAR were shown to be characterized by higher susceptibility to many viruses but were resistant to pathogens such as *Listeria monocytogenes *[43, 44]. Furthermore, genetic defects in interferon signaling pathway components cause severe forms of immunodeficiency [45, 46, 47, 48]. IFN-I binding to IFNAR initiates a signaling pathway leading to the induction of a group of interferon-stimulated genes (ISGs) [42, 49]. However, only few ISGs are directly involved in the development of the antiviral state. Many of them encode pattern recognition receptors (PRRs), which detect viral molecules and modulate signaling pathways or transcription factors increasing IFN production.

Some ISGs encode proteins exhibiting potential antiviral activity, including the proteins involved in cytoskeletal remodeling, apoptosis induction, and the regulation of posttranscriptional events (splicing, mRNA editing, RNA degradation, and different steps of protein synthesis), as well as the proteins involved in posttranslational modification [42]: for example, protein kinase R (PKR, also known as EIF2αK2), 2’-5′-oligoadenylate synthetase (2’-5′-OAS) and Mx GTPases (dynamin-like GTPases belonging to the Mx family), ribonuclease L (RNase L), ISG15 (15-kDa IFNinduced protein) have well-described antiviral functions. Mice carrying mutations or abnormalities in the key stages of signaling pathways activated by these proteins are characterized by increased susceptibility to viral infections [42].

PKR is an intracellular protein kinase that recognizes viral dsRNA, phosphorylates eIF2a (translation initiation factor 2A), and inhibits translation [39, 42, 50, 51]. PKR activation leads to the inhibition of protein synthesis in virus-infected cells, contributing to the rapid death of these cells and preventing the spread of infection.

2’-5′-OAS and ribonuclease L are components of the antiviral immune response of a cell. 2’-5′-OAS forms short oligoadenylates from ATP, which activate ribonuclease L, leading to viral RNA degradation. This process impedes virus replication and promotes destruction of the infected cells [42, 52].

ISG15 is a protein that modifies many cellular and viral targets via a process known as ISGylation. ISG15-induced ISGylation prevents the degradation of IRF3, an important transcription factor involved in the antiviral immune response [53]. Moreover, ISG15 indirectly stops virion release. ISG15 inhibits the ubiquitination of HIV Gag (group-specific antigen) and Tsg101 (tumor susceptibility gene 101 protein), which prevents viral release from the host cell. The interaction between the N-terminal domain of Tsg101 and the viral Gag protein is critical for the formation of new virus particles [52, 53, 54].

The Mx GTPase family plays an important role in the antiviral immune response. Human MxA interacts with the virus nucleocapsid and prevents viral transport, thus blocking replication. Furthermore, MxA inhibits viral transcription: thus, MxA was shown to bind to the PB2 subunit of influenza virus RNA polymerase and prevent viral genome transcription. This impedes viral replication and promotes the destruction of infected cells [52, 55, 56].

Antiviral functions were also reported for other ISGs: the adenosine deaminase (ADAR1) and APOBEC proteins; ISG20 exonuclease; TRIM (tripartite motif-containing) proteins such as TRIM19 (also known as PML), TRIM5a [57], Viperin (Cig5) [58]; and IFN-inducible translation regulators (IFIT1, IFIT2, and IFIT3) [42, 59, 60]. However, the functions of most of these ISGs remain poorly characterized to this day and their antiviral response mechanisms remain unknown.

Downregulating IFN expression or signaling of this cytokine by decreasing receptor expression or altering subsequent signaling may lead to the suppression of antiviral signaling pathways in different types of tumors. Furthermore, the antiviral response in cancer cells can be reduced by ISG deactivation: for example, downregulated PKR expression in tumor cells increases viral replication. In other cases, such as in low malignant tumors, PKR can remain active, which may have an impact on the effectiveness of oncolytic virotherapy [39]. Oncolytic viruses have a heightened specificity for cancer cells with a limited response to IFN, since in healthy cells viruses are eliminated through IFN-mediated responses [39].

**Vesicular stomatitis virus as an oncolytic**

VSV is a virus with a nonsegmented negative-sense RNA genome, belonging to the family *Rhabdoviridae*. The family *Rhabdoviridae *comprises more than 100 viruses, which infect both vertebrates and invertebrates, as well as plants [4]. There are eight major serotypes of VSVs: Indiana (VSVInd), New Jersey (VSVNJ), Cocal virus (COCV), Alagoas VSV (VSVala), Isfahan (ISFV), Chandipura (CHAV), Maraba, and Piry virus (PIRYV) [61, 62, 63, 64]. A VSV mostly affects livestock and is transmitted by direct contact through aerosols and fomites. In humans, VSV infections are usually asymptomatic. However, fever, chills, muscle pain, and nausea are observed in some cases [65]. Recombinant VSV (rVSV) is a promising vaccine vector, because its simple genome can accommodate multiple foreign genes; it neither undergoes recombination nor does it integrate the host cell DNA but achieves high titers (>109 plaque-forming units, PBU/mL [66]) in various cell types, which facilitates the production of virus-based drug. Moreover, VSV-based vaccines induce a potent cell-mediated and humoral immune response to abundantly expressed foreign antigens [67]. Furthermore, a very small percentage of people are seropositive for VSV [66].

There are several protocols for the assembly of recombinant VSVs [68, 69, 70, 71]; most of them involve transfection of mammalian cells with plasmids expressing the N, P, G, and L proteins of VSV VSV-based drugs still in their infancy, followed by coinfection of cells with viruses expressing the DNA-dependent T7 RNA polymerase (T7 RNA polymerase). In addition, protocols where an accessory plasmid also encoding T7 RNA polymerase is used during cell transfection [72], or VSV assembly occurs in genetically modified cell lines, have been published [71].

Most of the protocols describe methods for producing the VSV using the wild-type or modified vaccinia virus (VACV or VV) [70] to ensure more efficient translation of the VSV genes [68]. However, the assembly scheme involving cell transfection with five plasmids (the plasmid expressing the virus genome and four accessory plasmids expressing the N, P, G, and L proteins of VSV) and additional transduction of VV imposes a significant cellular burden and reduces the efficiency of virus assembly. Furthermore, one needs to take into account that the virus-based drugs used *in vivo *must not contain residues of VV or other viruses; so, there needs to be an additional step involving the purification of the resulting virusbased drug [71]. Therefore, other assembly techniques are recommended for producing drugs of high-purity grade and free of viral contamination.

Application of the accessory fifth plasmid expressing T7 RNA polymerase helps avoid drug contamination but can significantly reduce cell transfection efficiency. Successful virus assembly involves the simultaneous expression of six plasmids (the plasmid expressing the viral genome, the plasmid expressing T7 RNA polymerase, and four accessory plasmids expressing the N, P, G, and L proteins of VSV). However, not all of these plasmids can penetrate into cells in the amounts needed for virus assembly; furthermore, they impose a metabolic burden on cells.

Genetic modification of cell lines for the assembly of recombinant VSV seems to be the most practical way of virus assembly that requires no additional purification steps. Thus, Moroz et al. demonstrated that VSV could be efficiently assembled in the HEK293TN-T7 cell line expressing the T7 RNA polymerase gene and transfected with the plasmid expressing the viral genome and four accessory plasmids expressing the N, P, G, and L proteins of VSV.

Although there exist operational protocols for VSV assembly, it is necessary to continue searching for the most efficient assembly schemes that could be simpler and help one produce high-quality virus-based drugs.

The chance of using rVSV in many types of cancers, including prostate [6], skin [7], colon [8], pancreatic [9], and other types of cancer [10], is being considered. The VSV is a potent inducer of apoptosis in many types of cancer cells; it is very susceptible to the antiviral effects of IFN and, therefore, selectively replicates in cancer cells with defects in the IFN pathway [73]. Attenuated VSV strains were constructed to ensure heterologous gene expression, improved selectivity with respect to cancer cells, better cancer cell destruction rate, or enhanced antitumor immunity. In preclinical trials, recombinant VSV strains were found to be highly effective against a wide range of tumors [74, 75, 76]. Thirteen clinical trials to assess the effectiveness of VSV in different cancers are currently underway (https://www.clinicaltrials.gov/). Thus, rVSV expressing the human interferon beta (IFN-β) gene and rVSV expressing two supplementary genes (the *IFN-β* gene and the *TYRP1 *gene that encodes tyrosinaserelated protein 1 and is expressed in melanocytes), are currently in phase I clinical trials aiming to assess treatment of hepatocellular carcinoma (NCT01628640) and stage III/IV melanoma (NCT03865212), respectively.

**Methods for arming (editing) VSVs to enhance the effectiveness of VSV-based drugs**

The development of novel safe VSV strains is extremely important, since this virus has a broad tropism. Different genomic modifications are introduced into VSV in order to improve safety and clinical efficacy. There are several strategies for VSV attenuation: (1) limiting replication (e.g., using pseudotyped viruses with *G*-gene deletion [77]); (2) reducing the viral gene expression (e.g., moving the *N *gene from position 1 to position 4 in the genome [78, 79]); (3) inhibiting virus maturation (e.g., by truncating the C-terminus of the G protein [80]); and (4) ensuring a faster antiviral response of the host to attenuate viral replication, production and transmission by incorporating a mutation in the M protein (e.g., by amino acid deletion or substitution at position 51 [81, 82]).

Additional insertions into the virus genome are made to increase the effectiveness of VSV-based drugs [83]. Many genes are inserted into the genome to stimulate the immune response to the tumor (e.g., the genes encoding IL-12, GM-CSF, tyrosine kinase, CD40L, IL-15, etc. are inserted into the rVSV genome) [5, 84, 85, 86]. Thus, Shin et al. experimentally demonstrated that VSV-IL12 expressing the proinflammatory cytokine IL-12 has a direct cytotoxic effect in mice with squamous cell carcinoma (SCC) of the head and neck: they observed a reduced tumor volume and increased chances of animal survival [30, 86, 87]. MicroRNAs, short non-coding RNAs regulating gene expression by inhibiting the translation of target transcripts, were also used to modify VSVs in order to enhance selectivity and effectiveness. The microRNA expression profiles vary in different tissues and change along with progression of the disease, including cancer [88].

**Recombinant VSVs in clinical research**

The vesicular stomatitis virus has proved to be a highly effective oncolytic for treating a broad range of malignant tumors in a large number of preclinical studies [30, 83, 89]. Most of the clinical trials currently underway seek to evaluate the effectiveness and safety of VSV-hIFNβ-NIS carrying the human interferon-beta (IFNβ) gene for enhancing the selectivity of the oncolytic and the sodium iodide symporter (NIS) to control biodistribution of the virus. Clinical trials are underway for the VSV-GP154 and VSV-GP128 viruses, in which the *G *gene is replaced with the *GP *gene of the lymphocytic choriomeningitis virus (LCMV) in order to reduce the potential neurotoxicity of VSV. The VSV-hIFNβ-TYRP1 variant has been used in clinical trials as an agent against stage III/IV melanoma. Along with the gene encoding human IFNβ, the TYRP1 gene expressed in melanocytes was also inserted into its genome in order to increase the oncolytic selectivity of VSV-hIFNβ-TYRP1. Most clinical trials of VSV-based drugs are conducted in combination with various immunotherapeutic approaches.

The VSV is used for patients with a broad range of malignancies; patients with recurrent and metastatic solid tumors (colorectal cancer (NCT02923466, NCT04046445, and NCT04291105), melanoma (NCT03017820, NCT03865212, and NCT04291105), endometrial cancer (NCT03120624 and NCT03456908), head and neck cancer (NCT04291105), pancreatic cancer (NCT05846516), and other tumors (NCT05644509 and NCT01042379) are chosen the most often. Clinical trials involving patients with malignant lymphoma (NCT06508463 and NCT04046445) are also being conducted. Unfortunately, the results of these clinical trials are yet to be published.

**Obstacles to the application of virotherapy**

Despite the significant therapeutic potential of oncolytic viruses, there also exist many limitations that impede their use, such as the risk of a profound systemic immune response of the body; physical barriers in the tumor and barriers in the immunosuppressive tumor microenvironment (TME) and the challenges related to the delivery of virus particles and their replication in cancer cells; the choice of the optimal combination of oncolytic viruses and other drugs, the administration scheme and route; as well as challenges related to the production of virus-based drugs and maintaining a high titer of virus particles.

A viral platform should be carefully selected in order to minimize the risk of a systemic immune response of the body. The oncolytic properties of the viruses safest for humans, which include those belonging to the families *Adenoviridae*, *Herpesviridae*,* Poxviridae*, *Picornaviridae*, *Paramyxoviridae*,* Rhabdoviridae*, *Parvoviridae*, and *Reoviridae *[30], are being studied for this purpose. Capsid modification is used to solve the problems related to the delivery of oncolytic viruses to cancer cells and the insufficient specificity of delivery: it strengthens the binding of virus particles to the receptors responsible for penetration into target cells [90] and the deletion of viral genes needed for virus replication in normal cells. Thus, ONYX-015, an oncolytic adenovirus with deleted gene coding for the E1B protein, shows an increased ability for selective replication in tumors, since the modified virus cannot inactivate protein p53 in normal cells [91, 92]. Polyethylene glycol (PEG), poly-N-(2-hydroxypropyl)methacrylamide, thiol groups for attaching transferrin to capsid proteins, etc. are also used to modify oncolytic viruses [91, 92, 93, 94, 95].

Furthermore, a search for markers of susceptibility of cancer cells to this virus is underway [96] in order to improve the effectiveness of VSV for the treatment of malignant tumors, which will be discussed more thoroughly in the following sections.

**Table 1:** Changes in the expression of the EGFR and HER2 genes in cell lines characterized by different susceptibilities to VSV

Cell line	Level of EGFR and HER2 expression	Susceptibility to virotherapy with recombinant VSV	VSV serotype
HOS (osteosarcoma)	Upregulated [96]	High [96]	VSV strain Indiana
DBTRG-05MG (glioblastoma)	Downregulated	[96] Low [96]	VSV strain Indiana
U251MG (glioblastoma)	Upregulated [96]	High [96]	VSV strain Indiana
A172 (glioblastoma)	Upregulated [96]	High [96]	VSV strain Indiana
U87MG (glioblastoma)	Upregulated [101]	High [102]	rVSV-ΔM51
A375 (melanoma)	Downregulated [99]	High [103]	Wild-type VSV
A549	Upregulated [104]	Low [105]	VSV strain Indiana
HepG2	Upregulated [106]	High [107]	rVSV-GFP
SW982 (synovial sarcoma)	Upregulated	[108] Low [109]	rVSV-G/GFP
BxPC-3	Moderate [110]	High [111]	rVSV-ΔM51-GFP
AsPC-1	Downregulated [112]	Low [9, 111]	rVSV-ΔM51-GFP
Capan-1	Downregulated [113]	Low [9, 111]	rVSV-ΔM51-GFP
Panc-1	Downregulated [112]	Low [9, 111]	rVSV-ΔM51-GFP
MIA PaCa2	Moderate [112]	Low [9, 111]	rVSV-ΔM51-GFP
Capan-2	High [113]	Low [9, 111]	rVSV-ΔM51-GFP
T3M4	Moderate [114]	Low [9, 111]	rVSV-ΔM51-GFP
CFPAC	Upregulated [110]	High [9, 111]	rVSV-ΔM51-GFP
HPAC	Upregulated [110]	High [9, 111]	rVSV-ΔM51-GFP
HPDE	High [115]	High [9, 111]	rVSV-ΔM51-GFP
Hs766T	High [113]	High [9, 111]	rVSV-ΔM51-GFP

**Biomarkers of cancer cell resistance to rVSV virotherapy**

Defects in interferon pathways that are typical of cancer cells [97] make oncolytic viruses promising therapeutic agents, but tumors differ greatly in terms of their susceptibility to viruses. For example, *in vitro* experiments demonstrated that some cancer cell lines incubated with IFN-1 acquired resistance to VSV, while others remained susceptible to its cytopathic activity [11]. J. Noser et al. showed that the activated RAS/Raf1/MEK/ERK pathway plays a crucial role in the emergence of abnormalities in the antiviral response in cancer cells. In particular, they demonstrated that infection with VSV causes rapid death of the NIH 3T3 cell line stably expressing active RAS or Raf1 [98].

The search for biomarkers of the susceptibility of cancer cells to oncolytic viruses, and VSV in particular, revealed that the *EGFR *and *HER2 *genes are typically overexpressed in VSV-susceptible cell lines, unlike in resistant ones [96, 99] (*Table 1*). These findings suggest that activation of the EGFR/HER2 pathway and *HER2 *gene overexpression can be potential biomarkers of tumor vulnerability to VSV oncolytic therapy [100].

**Fig. 2:**
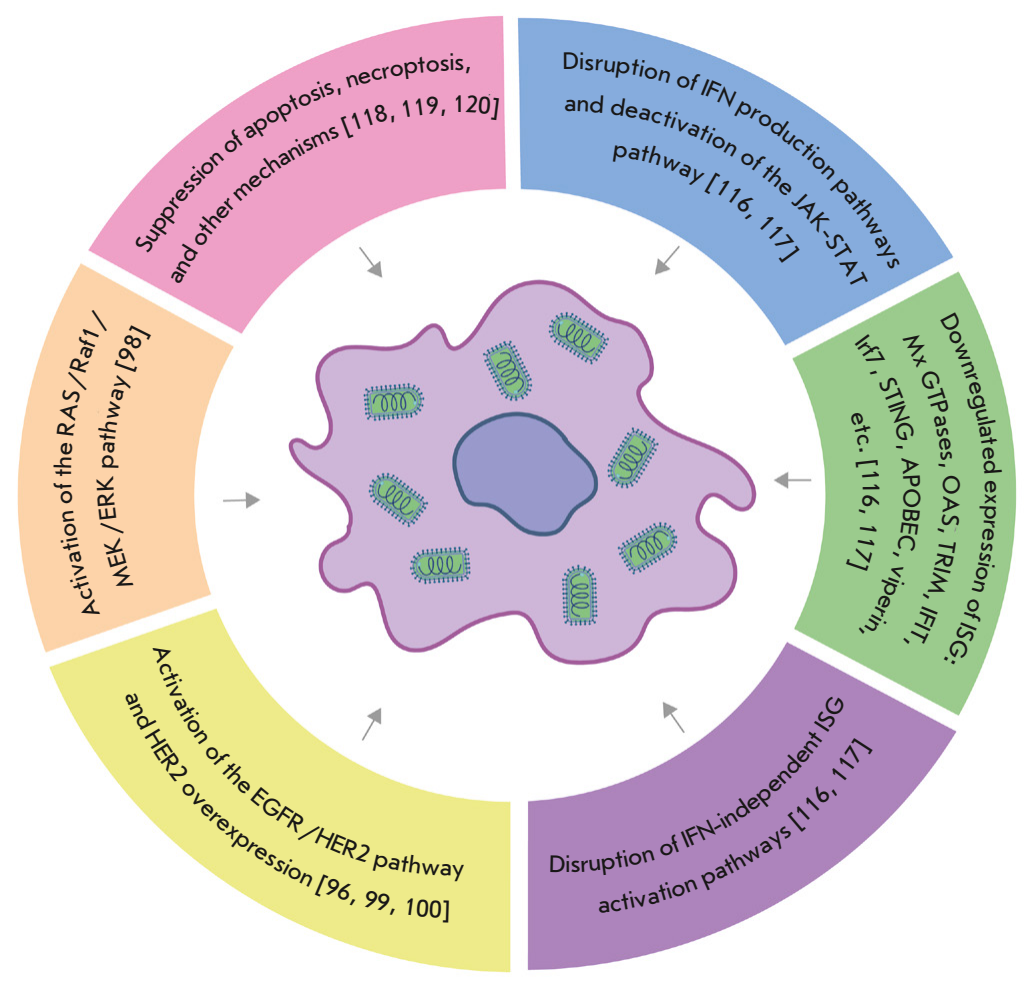
A cell susceptible to VSV therapy. Knowing the molecular mechanisms underlying the differences in the susceptibility of cancer cells to viruses is essential for elaborating approaches to cancer treatment, identifying biomarkers of susceptibility to specific oncolytic viruses, predicting the effectiveness of virotherapy in each individual patient [96], and improving the effectiveness of cancer treatment

Disturbances in the antiviral response in cancer cells, such as changes in IFN production pathways and deactivation of the JAK-STAT pathway, as well as reduced ISG production (Mx GTPase, OAS, TRIM, IFIT, Irf7, STING, APOBEC, viperin, etc.) [116, 117], may affect susceptibility to oncolytic viruses. The molecules involved in other mechanisms can also act as potential susceptibility markers. Thus, the hepatitis C virus (HCV) activates mitochondrial fission in the host cell, resulting in apoptosis inhibition and virus replication [118]. Inhibition of mitochondrial fission and mitophagy via the suppression of Drp1 (dynamin- related protein 1) led to reduced HCV replication and increased cellular resistance to viral infection [119]. Inhibition of necroptosis in cells was shown to enhance replication of the Zika virus (ZIKV) [120]. Meanwhile, downregulated RIPK3 expression can increase the susceptibility of cells to viral infection [121] (*Fig. 2*).

## CONCLUSIONS

VSV-based drugs are promising antitumor agents, but it still remains essential to search for novel molecules that, as they are integrated into the VSV, would enhance its lytic and immunostimulatory properties, thus increasing the effectiveness and safety of such drugs

Designing an effective VSV-based drug is complicated by the fact that some cancer cells are insusceptible to the virus, which may lead to poor therapy effectiveness in these types of malignant tumors. The effectiveness and safety of VSV-based drugs can be improved by incorporating mutations that increase the susceptibility of the virus to the cancer cells in the virus genome [81, 82], as well as by combining them with other oncolytic viruses, immunomodulators, CAR-T cell therapy agents, and conventional methods such as chemotherapy, surgery, and radiation therapy [5, 122, 123]. No universal markers for susceptibility to VSV virotherapy that would allow one to evaluate the effectiveness of the oncolytic in a specific tumor type and assess whether VSV virotherapy is suitable for a given patient have been identified thus far [96]. Therefore, more thorough research into the molecular mechanisms underlying the differences in the susceptibility of cancer cells to viruses, as well as the features of antiviral defense in cells in response to a VSV infection, is needed.

## Abbreviations

VSV: vesicular stomatitis virus
rVSV: recombinant vesicular stomatitis virus
ISG: interferon- stimulated gene
IFNs: interferons
PAMP: pathogen-associated molecular pattern
MHC: major histocompatibility complex
IFNAR: type I IFN receptor
PKR: protein kinase R
